# A cross-sectional study of dietary non-adherence and perceived barriers of people living with type 2 diabetes mellitus in a rural community in the Ashanti region of Ghana

**DOI:** 10.11604/pamj.2025.51.108.48179

**Published:** 2025-08-29

**Authors:** Ernestina Armah, Afriyie Kofi Addae, Emefa Jeshurun Nkonu, Manasseh Bannor Wireko, Michael Darko Ashaley, Samudeen Sani, Hendricks Jacobus, Marlise Van Staden, Isaac Kofi Owusu

**Affiliations:** 1 Department of Nursing and Midwifery, Christian Service University, Kumasi, Ghana,; 2 Department of Public Health, Nutrition Unit, New Edubiase Government Hospital, New Edubiase, Ghana,; 3 Department of Public Health, Withrow College, Agona-Asamang, Ashanti Region, Ghana,; 4 Department of Public Health, Nutrition Unit, Saint Martin Hospital, Manso, Ghana,; 5 Department of Theoretical and Applied Biology, Kwame Nkrumah University of Science and Technology, Kumasi, Ghana,; 6 Department of Biological Science Education, Akenten Appiah-Menka University of Skills Training and Entrepreneurial Development, Mampong-Ashanti, Ghana,; 7 Biostatistics Unit, Department of Surgery, Korle-Bu Teaching Hospital, Korle-Bu, Ghana,; 8 Department of Physiology and Environmental Health, University of Limpopo, Limpopo, South Africa,; 9 Department of Medicine, Kwame Nkrumah University of Science and Technology, Kumasi, Ghana,; 10 Directorate of Medicine, Komfo Anokye Teaching Hospital, Kumasi, Ghana

**Keywords:** Non-adherence, type 2 diabetes mellitus, perceived barriers, rural community

## Abstract

**Introduction:**

diabetes is undergoing an epidemiologic transition in sub-Saharan Africa, driven by factors such as the westernization of the African diet and changes in personal lifestyle. This transition of the African diet is seen more in urban than rural communities. Dietary counseling is subject to non-adherence because people living with type 2 diabetes mellitus (T2DM) are often counseled on diets that are usually difficult to come by in rural communities, and are unpalatable for some. This study, therefore, aimed to investigate dietary non-adherence and perceived barriers to the recommended diet among individuals living with T2DM in a rural community in Ghana.

**Methods:**

a descriptive cross-sectional survey design with a quantitative approach was used to assess the dietary adherence/non-adherence to dietary counseling of 208 T2DM participants receiving care at the new Edubiase government hospital. Dietary non-adherence was measured as an outcome variable against predictors such as socio-demographic factors, biological risk factors, duration of T2DM, presence of comorbidities (hypertension), and knowledge of T2DM.

**Results:**

females were the majority, 73.6% (n=153), and the average age was 58 (SD ±11.9). The prevalence of non-adherence was 39.42% (n=82). About 24.5% (n=51) of the participants ate fruits and vegetables 25% (n=52) every time in their main meal. A total of 43.3 % (n=90) of the participants were engaged in inappropriate dietary habits. The majority cited food unavailability 84.6% (n=176), inaccessibility 80.8% (n=164), and financial constraints 60.1% (n=125) as perceived barriers to dietary non-adherence. The majority, 65.9% (n=137) of the participants, indicated that food preferences and taste contribute to non-adherence to diabetic diets. Among participants who adhered to the recommended diet, females with tertiary education and T2DM onset of less than 5 years were associated with higher adherence (females -aOR = 7.33, 95% CI = 2.33-22.99, P = .001, aOR = 3.69, 95% CI = 1.53-8.88, P = .001). Participants who did not use complementary herbal remedies had higher odds of adhering to dietary recommendations compared to those who used herbal remedies (aOR = 6.53, 95% CI = 2.34-18.23, P = .001). Among participants who were not adhering to recommended diets were those who had poor knowledge of T2DM management and a high Waist-to-Hip Ratio (WHR) (aOR = 7.97, 95% CI = 2.61-24.31, P = .001, aOR = 0.06, 95% CI = 0.01-0.33, P = .001).

**Conclusion:**

the high rates of non-compliance with dietary recommendations underscore the need for comprehensive and targeted interventions to address the multifaceted barriers faced by persons living with T2DM in rural communities.

## Introduction

The prevalence of T2DM poses significant challenges to individuals, families, and societies globally [1,2]. Dietary management plays a crucial role in effective T2DM management [3]. The Mediterranean Diet (MedDiet) has shown potential benefits for type 2 diabetes (T2D) management and prevention. Studies indicate that the MedDiet can positively influence cardiovascular risk factors, blood pressure, lipid profiles, insulin resistance, inflammation, and glucose metabolism in T2D patients [4,5]. The MedDiet's components, including vegetables, fruits, olive oil, and fish, may contribute to T2D-related mechanisms through anti-inflammatory and antioxidant properties, as well as effects on gut microbiota [4,5]. Approximately 9.3% of adults are affected by type 2 diabetes globally [2]. This translates to around 463 million people. In Africa, about 19.8 million people are affected by diabetes [2], with type 2 accounting for most of the cases [6]. It is unfortunate to note that 69.2% of cases go without being diagnosed as a result of inadequate health systems [7-9]. Type 2 diabetes constitutes 90% of all diabetic cases in Africa [9-11]. In the case of sub-Saharan Africa, there is a regional prevalence rate of 5.1%, ranging from 2.6% to 22.5% [12]. The disease accounts for 85-95% of diabetes cases in sub-Saharan Africa, and 62.5% remain undiagnosed [12].

In Ghana, the prevalence of diabetes falls between 2.8% and 3.95%, which is below the sub-Saharan average of 4.5% [13]. However, a number of studies report higher estimates, such as 39.8% in the Western region and 25.2% in the Ashanti region [14,15]. In Ghana, adherence to a MedDiet is the gold standard for dietitians and health professionals. It is characterized by a high intake of whole foods such as seeds, vegetables, fruits, legumes, and whole grains. Some studies have underscored the complexities surrounding dietary adherence in rural settings, where socioeconomic disparities, cultural preferences, and limited access to healthcare services exacerbate the difficulties faced by individuals in managing their condition [16,17]. In the Ghanaian context, particularly in rural districts of the Ashanti region, the extended family system and communal eating practices present additional barriers to adhering to recommended dietary restrictions. The high cost of fresh produce and whole grains, coupled with the widespread availability of processed and unhealthy foods, further complicates efforts to maintain a balanced diet [18]. These challenges highlight the need for comprehensive and targeted interventions that take into account the multifaceted barriers faced by individuals with T2DM in these settings [19].

This study, therefore, aimed at exploring the dietary non-adherence and perceived barriers to the recommended diet among people living with T2DM in a rural community in Ghana. By understanding these dynamics, the study seeks to inform the development of context-specific suggestions to improve dietary adherence and enhance the overall management of T2DM in similar rural settings.

## Methods

**Study design and setting:** a descriptive cross-sectional research design with a quantitative approach was used to assess dietary non-adherence and perceived barriers to the recommended diet among 208 individuals living with T2DM. The study was conducted at New Edubiase Government Hospital in the Adansi South district of the Ashanti region, Ghana. New Edubiase, the district capital, is located in the region´s southernmost area and shares borders with Atobiase to the south, Amudurase to the north, Menang to the west, and Bronikrom to the east. With its strategic location and accessible road networks, the hospital serves as a key referral center for public and private clinics and hospitals in neighboring towns and villages. The hospital includes a specialized diabetes unit for persons with T2DM, along with surgical, dental, physiotherapy, and eye care departments, providing comprehensive services to both inpatients and outpatients. Notably, facility dieticians, health promotion officers, and community nutrition officers are essential in counseling these patients on diet, empowering them to make informed dietary decisions and choices that can significantly impact their condition.

**Study population:** the study population consisted of adult individuals living with T2DM who visited the outpatient departments (OPDs) of New Edubiase Government Hospital regularly. With an eligible large population, a systematic sampling technique was used by selecting every 4th patient to ensure equal inclusion.

**Inclusion criteria:** individuals diagnosed with T2DM, who were above eighteen (18) years old, visited the chronic care clinic of New Edubiase Government Hospital regularly, and were prepared to participate in the survey.

**Exclusion criteria:** i) individuals living with T2DM who were not mentally stable; ii) individuals who were newly diagnosed with type T2DM within the past month; iii) individuals younger than eighteen (18) years; iv) pregnant and lactating women, as a result of their specific dietary needs; v) individuals who were on admission and required medical care and monitoring were excluded from this study; vi) persons living with T2DM who had undergone major surgical procedures within the last six months, as this may affect their dietary needs and overall health; vii) individuals with co-existing medical conditions that required specific dietary interventions unrelated toT2DM, such as chronic kidney disease or gastrointestinal disorders.

**Sampling technique and sample size:** a convenience sampling method was used to select the study participants. The sample size was determined using Cochran's formula for a single population proportion [20]. This is based on statistical principles and considerations related to precision, confidence level, and the prevalence of the variable of interest (dietary adherence). Using a prevalence of dietary adherence of 14.29% [21] and a 95% level of significance, with a margin of error of 5%, the sample size was calculated.


$$n=\frac{Z^{2}p}{E^{2}}$$

n = the desired sample size, E= margin of error at 5% =0.05, Z = Confidence interval (Cl) of 95% = 1.96, P = prevalence of dietary adherence 14.29% = 0.1429, q= (1-p) = the proportion non-dietary adherence = (1- 0.1429) = 0.8571. Therefore:


$$\begin{array}{ccc} n & = & \frac{1.96^{2}0.8571}{0.05^{2}}\\ n & = & \frac{0.4705176}{0.0025} \end{array}$$

n = 188.207. Using 10% as the attrition rate, the final sample size was 208 for the study.

**Data collection:** structured questionnaires were administered to collect demographic data, dietary habits, and factors influencing dietary adherence. Knowledge was assessed through a composite variable of five items, with each correct response scored as 1 and incorrect responses as 0. The knowledge instrument achieved a content validity index (CVI) score of 0.85, established by a panel of five (5) experts who rated each survey item on a scale from '1' (not relevant) to '5' (highly relevant). The Item-Level CVI (I-CVI) was calculated as the proportion of experts who rated items as 4 or 5, and the overall CVI was determined by averaging I-CVIs across all items. Knowledge was scored poorly if the mean score was between 1 and 8, average when it was 9-12, and good when participants scored 13-18. Dietary adherence was measured using the perceived dietary adherence questionnaire (PDAQ) [22], which is a nine-item questionnaire, developed by Asaad *et al*. [22] and modified to fit the Ghanaian context, with each item offering two response options: "Yes" (scored 1) and "No" (scored 0).

### Definitions

**Outcome variable:** dietary non-adherence: this was the primary outcome or response variable in the study. Dietary adherence was defined as eating according to dietary recommendations by health professionals. Using the median score, it was dichotomized as either dietary adherence or non-adherence. For analysis, it was scored as 0 = non-adherence, 1 = adherence.

**Predictor variables:** the predictor variables of this study were: i) background variables (socio-demographic factors): gender, employment status, income level, history of T2DM; ii) biological risk factors (BMI, WHR); iii) duration of T2DM; iv) presence of comorbidity (hypertension); v) knowledge of T2DM.

**Background characteristics of study participants:** they consisted of age and gender. Age was categorized a priori using existing studies on dietary adherence in the sub-Saharan Africa. Gender was measured as a binary variable (male and female).

**Biological risk factors of study participants:** they included body mass index (BMI) and waist-to-hip ratio (WHR). The BMI was also calculated as the weight of participants in kilograms divided by the square of their height in meters. BMI was categorized as underweight (<18.5), normal (18.5-24.9), overweight (25-29), and obese (30-39.9) [23]. Anthropometric measurements: weight, height, hip circumference, and waist circumference were taken from study participants. Other risk factors included in the analysis were family history of T2DM, educational status, and employment status.

**Presence of comorbidity (hypertension):** participants' history of hypertension was obtained from the clinical health records. This was done by the confirmation of participants' diagnosis of hypertension and the use of anti-hypertensive drugs from the participants' folders. For analysis, hypertension was considered as either hypertensive or non-hypertensive.

**Knowledge of T2DM:** knowledge was assessed using a total score ranging from 1 to 18. The classification of knowledge levels into 'poor', 'average', and 'good' was based on the interquartile range (IQR) of the score distribution [24]. Scores below the first quartile (Q1) were categorized as ‘poor’, scores within the interquartile range (Q1 to Q3) were categorized as ‘average´, and scores above the third quartile (Q3) were categorized as 'good'.

**Data analysis:** data obtained from the structured questionnaires were analyzed using Statistical Package for the Social Sciences (SPSS) software, version 27. The raw data of the questionnaires were cleaned to eliminate any incomplete, erroneous, or inconsistent responses, thereby ensuring only valid data were included in the analysis. The data analysis procedure involved descriptive statistics to summarize participants' background characteristics, adherence levels, lifestyle habits, and perceived barriers to dietary adherence. Frequency distributions and percentages were used to describe categorical variables, while means and standard deviations characterized continuous variables. Adherence contained 10 items, which were a binary response variable. The Cronbach's alpha test was 0.79, showing a high reliability and consistency measure. Variables were selected for inclusion in the multivariable logistic regression model based on statistical significance from the univariable analysis, with variables demonstrating p-values ≤ .05 being considered for inclusion. Based on the univariable screening, six variables met the significance criterion and were included in the multivariable model: gender (P = .021), T2DM duration (P = .022), knowledge level (P = .0001), BMI (P = .030 for normal weight), waist-to-hip ratio (P = .032), and herbal use (P = .043). Variables that did not meet the significance threshold were excluded from the multivariable model, including age (all categories P > .05), non-significant educational level categories, marital status (P = .710), family history of T2DM (P = .70), and treatment type (P > .05). A multinomial logistic regression analysis was used to determine associations between background factors (e.g., gender, education level, T2DM duration) and dietary adherence, adjusted for confounders. Adherence odds ratios were calculated, with variables such as gender, educational status, T2DM duration, and dietary knowledge assessed as significant predictors. This comprehensive approach identified factors that influenced dietary adherence and non-adherence.

**Ethics approval and consent to participate:** ethical clearance for the study was secured from the Committee on Human Research, Publications, and Ethics (CHRPE) with the number CHRPE/AP/338/24 before the commencement of the study. Permission was obtained from the district health directorates and the management of New Edubiase Government Hospital. Written consent was sought from participants after explaining the study and its relevance to them. The study adhered to the protocols of the Helsinki Declaration.

## Results

**General characteristics of the study population:** the average age was 58 years (SD ±11.9). From Table 1, 40% (n=85) of participants had basic education, while 20.7% (n=43) had tertiary education. Half of the participants (50%) were self-employed, 18.3% (n=38) were unemployed, and 14.9% (n=31) were retired (Table 1). The majority, 65.4% (n=136) of participants reported having a family history of T2DM (Table 1). Almost half, 45.2% (n=94) of the participants attended regular counseling sessions with the hospital dietician or the person responsible for counselling, 41.3% (n=86) had attended only once (see table 1). The majority of participants, 69.2% (n=144), were on oral hypoglycemic agents and dietary counseling. Finally, 29.8% (n=62) indicated that they used herbal remedies (Table 1).

**Table 1:** demographic and clinical characteristics of participants recruited from New Edubiase Government Hospital (Ghana), from June 2024 to August 2024 (N=208)

Variables	Freq (n)	Percent (%)
Age ±SD	58±11.9	
**Age groups**		
20-30 yrs	6	2.9
31-40 yrs	7	3.4
41-50 yrs	35	16.8
51-60 yrs	68	32.7
61-70 yrs	65	31.3
71+ yrs	27	13.0
**Gender**		
Female	153	73.6
Male	55	26.4
**Marital status**		
Single	9	4.3
Married	130	62.5
Divorce	35	16.8
Widowed	34	16.3
**Education level**		
No formal	54	26.0
Primary	85	40.8
Secondary school	26	12.5
Tertiary	43	20.7
**Employment status**		
Private employment	16	7.7
Public sector	19	9.1
Retired	31	14.9
Self-employed	104	50.0
Unemployed	38	18.3
**Family history of T2DM**		
No	72	34.6
Yes	136	65.4
History of hypertension		
Non-hypertension	54	26.0
Hypertension	154	74.0
**Counseling sessions with the hospital dietician**		
Attended once	86	41.3
Attend regular	94	45.2
Never attended	28	13.5
Treatment		
Diet + Oral agents	144	69.2
Diet + Insulin	12	5.8
Diet + Oral agents + Insulin	52	25.0
**Use of herbal medication**		
No	146	70.2
Yes	62	29.8

A significant proportion, 55.6% (n=115) of participants admitted to occasionally forgetting to follow the recommended dietary guidelines for T2DM management (Table 2). Notably, 69.3% (n=142) of participants indicated that they were sometimes forced to stop following their dietary plan when they traveled or left home (Table 2).

**Table 2:** adherence to Diabetes Mellitus (DM), dietary plan among participants recruited from New Edubiase Government Hospital (Ghana), from June 2024 to August 2024 (N=208)

Variable	Freq (n)	Percent (%)
**Do you sometimes forget to follow the recommended dietary approach for DM?**		
No	92	44.4
Yes	115	55.6
**Over the past two weeks, were there any days when you did not follow your dietary plan properly?**		
No	107	51.4
Yes	101	48.6
**Did you miss the proper dietary plan yesterday?**		
No	163	78.4
Yes	45	21.6
**Have you ever stopped following the recommended dietary plan without telling your doctor because you felt unnecessary to do so?**		
No	109	52.9
Yes	97	47.1
**When you feel like your DM is under control, do you sometimes stop following your dietary plan?**		
No	133	64.3
Yes	74	35.7
**When you travel or leave home, are you sometimes forced to stop following your dietary plan?**		
No	63	30.7
Yes	142	69.3
**Do you ever feel pressured to stick to your dietary plan?**		
No	95	45.9
Yes	112	54.1
**Did you have feelings of dietary deprivation?**		
No	94	45.2
Yes	114	54.8
**Do you forget to include fruits and vegetables in your dietary plan?**		
No	111	53.6
Yes	96	46.4
**Do you forget to cut down butter and fat intake in your food?**		
No	141	68.4
Yes	65	31.6

Participants who were females, 79.4% (n=100), married 63.5% (n=80), employed, 67.5% (n=85), diagnosed with T2DM less than five (5) years ago, were more likely to adhere to dietary recommendations than their respective counterparts (Table 3). In Table 4, 45.7% (n=95) of participants reported never exercising (Table 4). On consumption of condiments and spices, 60.6% (n=126) of participants used them once a month or less (Table 4). Lastly, on fried food consumption, 41.3% (n=86) reported eating fried foods once a month or less (Table 4). Out of all the participants, 62.5% (n=130) reported using sweeteners once a month or less (Table 5). Relative fruit consumption, 30.3% (n=63) of participants reported eating fruit less than once a week (Table 5). In terms of vegetable intake, 24.5% (n=51) reported eating vegetables less than once a week (Table 5). On protein consumption, 13.9% (n=29) of participants reported eating protein sources such as fish, meat, eggs, legumes, and leafy greens once a month or less (Table 5).

**Table 3:** background characteristics of corresponding and adherence of participants recruited from New Edubiase Government Hospital (Ghana), from June 2024 to August 2024 (N=208)

Variable	Non-Adherence N (%)	Adherence N (%)	Percentage of non-adherence within groups (%)
**Age**	-	-	-
20-30 yrs	2 (2.44)	4 (3.17))	33.33
31-40 yrs	4 (4.88)	3 (2.38)	57.14
41-50 yrs	16 (19.51)	19 (15.08)	45.71
51-60 yrs	23 (28.05)	45 (35.71)	33.82
61-70 yrs	22 (26.83)	43 (34.14)	33.85
71+ yrs	15 (18.29)	12 (9.52)	44.4
**Total**	82 (100.00)	126(100.00)	39.42
**Gender**	-	-	-
Female	53 (64.63)	100 (79.36)	34.64
Male	29 (35.37)	26 (20.64)	52.72
**Total**	82 (100.00)	126(100.00)	39.42
**Marital status**	-	-	-
Married	50 (60.97)	80 (63.49)	38.46
Single	32 (39.03)	46 (36.51)	41.06
**Total**	82 (100.00)	126 (100.00)	39.42
Employment status	-	-	-
Unemployed	28 (34.15)	41 (32.54)	40.57
Employed	54 (65.85)	85 (67.46)	38.85
**Total**	82 (100.00)	126 (100.00)	39.42
Daily income	-	-	-
<= Ghc 100	46 (59.74)	52 (53.1)	46.46
Ghc 101-299	16 (20.78)	39 (70.9)	29.09
<=Ghc 300	15 (19.48)	31 (67.4)	32.61
**Total**	77 (100.00)a	122 (61.3)b	38.69
**T2DM duration**	-	-	-
=<5 yrs	29 (35.36)	65 (51.59)	30.85
>5 years	53 (64.64)	61 (48.41)	46.49
**Total**	82 (100.00)	126 (100.00)	39.42
**BMI**	-	-	-
Underweight	16 (19.51)	38 (30.16)	29.62
Normal	50 (60.97)	57 (45.23)	46.73
Overweight	12 (14.63)	26 (20.63)	31.57
Obese	4 (4.88)	5 (3.97)	44.40
**Total**	82 (100)	126 (100)	39.42
**WHR**	-	-	-
Normal	35 (42.68)	34 (26.98)	50.72
Abdominal obesity	47 (57.32)	92 (73.02)	33.81
**Total**	82 (100.00)	126 (100.00)	39.42
**Knowledge**	-	-	-
Poor	22 (26.83)	58 (46.03)	27.50
Average	30 (36.59)	43 (34.13)	41.09
Good	30 (36.59)	25 (19.84)	54.54
**Total**	82 (100.00)	126 (100.00)	39.42
**Complementary herbal product**	-	-	-
No	51 (62.19)	95 (75.40)	34.93
Yes	31 (37.81)	31 (24.60)	50.00
**Total**	82 (100.00)	126 (100.00)	39.42
**Overall adherence**	-	-	-
Adherence	126 (60.58%)	-	-
Non-adherence	82 (39.42%)	-	-
**5 missing; b- 4 missing**			

**Table 4:** lifestyle and dietary habits survey of participants, recruited from New Edubiase Government Hospital (Ghana), from June 2024 to August 2024 (N=208)

Variable	Freq (n)	Percent (%)
**How many days do you exercise in a week?**		
Never	95	45.7
1 to 2 times a week	60	28.8
3 to 4 times a week	28	13.5
5 to 6 times a week	2	1.0
Daily	23	11.1
**How often do you eat meals in a day?**		
5 times	22	10.6
6 times	53	25.5
>6 times	133	63.9
**How often do you consume condiments?**		
Once a month or less	126	60.6
2 to 3 times a month	22	10.6
1 to 2 times a week	24	11.5
3 to 6 times a week	8	3.8
At least once daily	28	13.5
**How often do you drink sweetened beverages?**		
Once a month or less	150	72.1
2 to 3 times a month	19	9.1
1 to 2 times a week	19	9.1
3 to 6 times a week	10	4.8
At least once daily	10	4.8
**How often do you eat sweets?**		
Once a month or less	157	75.5
2 to 3 times a month	14	6.7
1 to 2 times a week	18	8.7
3 to 6 times a week	6	2.9
At least once daily	13	6.3
**How often do you eat fried foods?**		
Once a month or less	86	41.3
2 to 3 times a month	22	10.6
1 to 2 times a week	54	26.0
3 to 6 times a week	22	10.6
At least once daily	24	11.5

**Table 5:** dietary habits and influencing factors among participants recruited from New Edubiase Government Hospital (Ghana), from June 2024 to August 2024 (N=208)

Variable	Freq (n)	Percent (%)
**How often do you consume sugar and honey?**	-	-
Once a month or less	130	62.5
2 to 3 times a month	21	10.1
1 to 2 times a week	26	12.5
3 to 6 times a week	6	2.9
At least once daily	25	12.0
**How often do you eat fruit?**	-	-
Less than once a week	63	30.3
1 time a week	54	26.0
1 to 2 times a week	22	10.6
At least once a day	18	8.7
Every time in the main diet	51	24.5
**How often do you eat vegetables?**	-	-
Less than once a week	51	24.5
1 time a week	26	12.5
1 to 2 times a week	66	31.7
At least once a day	13	6.3
Every time in the main diet	52	25.0
**How often do you eat Protein (animal &plant)?**	-	-
Once a month or less	29	13.9
2 to 3 times a month	24	11.5
1 to 2 times a week	38	18.3
3 to 6 times a week	42	20.2
At least once daily	75	36.1
**How often do you eat out of the house?**	-	-
Less than 1 time in a month	114	54.8
1 time in a month	57	27.4
2 times in a month	21	10.1
More than once a week	16	7.7
**Inappropriate dietary habits**	-	-
No	118	56.7
Yes	90	43.3
**Financial constraints**	-	-
No	83	39.9
Yes	125	60.1
**Gathering with friends and family**	-	-
No	99	47.6
Yes	109	52.4
**Eating outside home**	-	-
No	90	43.9
Yes	115	56.1
**Food accessibility**	-	-
No	39	19.2
Yes	164	80.8
**Food availability**	-	-
No	32	15.4
Yes	176	84.6
**Time constraints**	-	-
No	98	47.3
Yes	109	52.7
**Religious and cultural beliefs**	-	-
No	107	53.0
Yes	95	47.0
**Food preferences and taste**	-	-
No	71	34.1
Yes	137	65.9

Factors cited for non-adherence included inappropriate dietary habits, 56.7% (n=118), financial 60.1% (n=125), eating outside the home 56.1 % (n=115), food unavailability 84.6% (n=176), and food preference and taste, 65.9% (n=137) (Table 6).

**Table 6:** univariable regression analysis showing odds of dietary non-adherence among participants recruited from New Edubiase Government Hospital (Ghana), from June 2024 to August 2024 (N=208)

Background variables	Unadjusted ORs (95% CI)	P-value	AOR (95% C.I.)	P-value
**Age (years)**	-	-	-	-
20-30	1	-	-	-
31-40	0.375(0.04-3.61)	0.396	0.41(0.02- 6.12)	0.52
41-50	0.594(0.10-3.68)	0.575	0.28(0.02- 2.80)	0.29
51-60	0.978(0.17-5.74)	0.981	0.90(0.10- 8.16)	0.93
61-70	0.977(0.17-5.76)	0.980	1.02(0.12- 8.92)	0.99
Above 70	0.400(0.06-2.57)	0.334	1.17(0.11- 12.40)	0.89
**Gender**	-	-	-	-
Male	1	-	-	-
Female	2.1(1.13-3.93)	0.021	7.325(2.334 - 22.985)	0.001
**Educational level**	-	-	-	-
No formal Education	1	-	-	-
Tertiary	1.30(0.54-3.10)	0.561	9.01(2.28- 35.52)	0.01
Secondary	0.31(0.12-0.83)	0.190	0.24(0.06- 0.96)	0.04
Basic	0.68(0.33-1.39)	0.291	0.65(0.24- 1.75)	0.40
**T2DM duration (years)**	-	-	-	-
<5	1	-	-	-
>5	0.51(0.29-0.91)	0.022	0.27(0.11- 0.65)	0.004
**Knowledge**	-	-	-	-
Poor	1	-	-	-
Average	0.54(0.28-1.08)	0.081	0.91(0.35- 2.34)	0.842
Good	0.32(0.15-0.6)	0.000	0.13(0.04- 0.38)	0.001
**BMI**	-	-	-	-
Underweight	1	-	-	-
Normal	0.49(0.24-0.96)	0.030	0.18(0.07- 0.49)	0.001
Overweight	0.91(0.37-2.24)	0.840	0.25(0.07- 0.91)	0.036
Obese	0.53(0.13-2.22)	0.380	0.14(0.02- 1.05)	0.056
**WHR**	-	-	-	-
Normal	1	-	-	-
Abdominal obesity	0.25(.07-0.89)	0.032	16.63(2.99- 92.47)	0.001
**Herbal use**	-	-	-	-
No	1			
Yes	0.54(0.29-0.98)	0.043	0.15(0.05- 0.43)	0.01
**Marital status**	-	-	-	-
Single	1	-	-	-
Married	1.11(0.63-1.97)	0.710	-	-
**Family history of T2DM**	-	-	-	-
Yes	1	-	-	-
No	1.13(0.63-2.04)	0.70		
**Treatment**		-	-	-
Diet + Oral agents	1	-	-	-
Diet + Insulin	4.54(0.98-21.01)	0.12	-	-
Diet + Oral agents + Insulin	1.34(0.69-2.61)	-	-	-

**Predictors of dietary non-adherence among respondents:** gender was a significant factor, with males being less likely to adhere (non-adhere) to dietary recommendations compared to females (aOR = 7.33, 95% CI = 2.33-22.99, P = .001) (Table 7). Similarly, individuals with no formal education were significantly more likely to be non-adherent compared to those with tertiary education (aOR = 9.01, 95% CI = 2.28-35.52, P = .001) (Table 7). Participants who had been diagnosed with T2DM for five years or more had lower odds of adhering to dietary recommendations than those with more recent diagnoses (aOR = 3.69, 95% CI = 1.53-8.88, P = .001) (Table 7). Poor knowledge of T2DM management was a significant predictor of non-adherence (aOR = 7.97, 95% CI = 2.61-24.31, P = .001) (Table 7). In addition, individuals with high Waist-to-Hip Ratio (WHR) were significantly more likely to be non-adherent (aOR = 0.06, 95% CI = 0.01-0.33, P = .001) (Table 7). The use of complementary herbal medications was associated with significantly non-adherence (aOR = 6.53, 95% CI = 2.34-18.23, P = .001) (Table 7).

**Table 7:** predictors of dietary non-adherence among participants recruited from New Edubiase Government Hospital (Ghana), from June 2024 to August 2024 (N=208)

Background variables	S.E.	Wald	Sig.	aOR	95% C.I.	
**Age (years)**	-	-	-	-	-	-
20-30	Ref	Ref	Ref	Ref	Ref	Ref
31-40	1.40	0.42	.52	0.41	0.02	6.12
41-50	1.17	1.17	.29	0.28	0.02	2.80
51-60	1.12	0.10	.93	0.90	0.10	8.16
61-70	1.11	0.00	.99	1.02	0.12	8.92
Above 70	1.21	0.02	.89	1.17	0.11	12.40
**Gender**	-	-	-	-	-	-
Male	Ref	Ref	Ref	Ref	Ref	Ref
Female	0.583	11.647	.001	7.325	2.334	22.985
**Educational level**	-	-	-	-	-	-
No formal education	Ref	Ref	Ref	Ref	Ref	Ref
Tertiary	0.70	9.86	.01	9.01	2.28	35.52
Secondary	0.71	4.08	.04	0.24	0.06	0.96
Basic	0.50	0.72	.40	0.65	0.24	1.75
**T2DM duration (years)**	-	-	-	-	-	-
<5	Ref	Ref	Ref	Ref	Ref	Ref
>5	0.45	8.50	.004	0.27	0.11	0.65
**Knowledge**	-	-	-	-	-	-
Poor	Ref	Ref	Ref	Ref	Ref	Ref
Average	0.48	0.04	.842	0.91	0.35	2.34
Good	0.57	13.30	.001	0.13	0.04	0.38
**BMI**	-	-	-	-	-	-
Underweight	Ref	Ref	Ref	Ref	Ref	Ref
Normal	0.51	11.26	.001	0.18	0.07	0.49
Overweight	0.66	4.40	.036	0.25	0.07	0.91
Obese	1.02	3.67	.056	0.14	0.02	1.05
**WHR**	-	-	-	-	-	-
Normal	Ref	Ref	Ref	Ref	Ref	Ref
Abdominal obesity	0.88	10.32	.001	16.63	2.99	92.47
**Complimentary herbal use**	-	-	-	-	-	-
No	Ref	Ref	Ref	Ref	Ref	Ref
Yes	0.52	12.82	.01	0.15	0.05	0.43

BMI= body mass index, WHR= waist-hip ratio, SE=Standard Error, CI=Confidence Interval

## Discussion

This study aimed to explore the level of dietary adherence and factors influencing adherence among persons living with T2DM in a rural district in the Ashanti region of Ghana. The findings revealed a significant prevalence of dietary non-adherence (39.35%) among participants, with diversity in adherence patterns observed. Key predictors such as gender, education level, duration of T2DM, body mass index (BMI), and comorbidities significantly influenced dietary non-adherence. Furthermore, food availability and accessibility, cultural influences, and the utilization of complementary herbal products emerged as significant barriers to adherence. This research emphasizes the intricate interactions of variables affecting dietary adherence among individuals with T2DM in Ghana, underscoring the necessity for tailored interventions and comprehensive strategies to facilitate optimal disease management. This study in Ghana found that 39.35% of individuals with T2DM struggle with dietary adherence, despite efforts to improve management [25,26]. This lack of adherence increases the risk of complications, worsens health outcomes, and burdens the healthcare system, negatively impacting the quality of life of diabetics and posing a significant public health challenge. Variability in adherence patterns is evident, with only 21.6% consistently following guidelines, 55.8% reporting occasional lapses, and 22.6% exhibiting poor adherence. In Ethiopia, a T2DM sample showed a 39.2% adherence rate, supporting the research findings [18]. The study also highlights the debate between dieticians and clients regarding the appropriate use of traditional or cultural food ingredients.

The significant correlation between dietary adherence and gender, with female participants exhibiting higher adherence rates (60.6%) compared to their male counterparts, is consistent with existing literature. This is likely due to cultural and social factors, where women are more responsible for meal planning and preparation, leading to better adherence. This highlights the need for gender-specific interventions to improve dietary adherence in individuals with T2DM. A study by Hendrychova *et al*. [27] confirmed that gender significantly influences dietary adherence among T2DM patients, with women generally adhering better due to household responsibilities and spousal support.

Dietary adherence (P<.001) was significantly influenced by education level, with higher education levels leading to improved adherence. This aligns with a study by Mogre *et al*. [18], which revealed that educational levels significantly influence T2DM self-care behaviors, such as diet, exercise, and foot care. Prior research highlights the importance of health literacy in managing chronic diseases. Higher education levels enhance health literacy, enabling patients to comprehend the importance of dietary adherence and its long-term consequences. Health literacy is crucial for chronic disease management, as it improves medication adherence and treatment regimens [6,28], preventing complications and enhancing health outcomes. Targeted interventions should help patients with lower education levels understand and implement dietary recommendations.

The duration of T2DM significantly impacts dietary adherence (P<.01), with non-adherence observed in those managing the condition for over five years. Some studies show a negative correlation, while others show no relationship. A study found a positive correlation between T2DM duration and hypoglycemia fear, leading to decreased treatment adherence [29]. However, a non-elderly study found lower dietary adherence in non-elderly individuals with T2D, contradicting the belief that longer disease duration leads to poorer adherence. Non-elderly individuals with T2DM may have lower dietary adherence due to complacency or increased complexity of managing the disease. Long-term management strategies, including continuous education and regular follow-ups, can help newer individuals develop the skills needed for effective dietary management.

Body mass index (BMI) was also significantly associated with dietary adherence, with obese individuals living with T2DM showing lower adherence rates compared with normal BMI. Obesity-related factors, like increased appetite, physical limitations, and metabolic disturbances, contribute to poor dietary adherence. This finding is consistent with research conducted by Chappidi *et al*. [30] that demonstrated that approximately 63% of participants in the study partook in unrestricted dietary consumption, potentially leading to increased body weight and suboptimal glycemic regulation. Additionally, comorbidities like hypertension and cardiovascular disease complicate dietary adherence. Healthcare providers should consider BMI and comorbidities when developing individualized dietary plans. Behavioral interventions like cognitive-behavioral therapy and motivational interviewing can help overcome perceived barriers and promote better adherence in T2DM patients.

Food availability and accessibility emerged as significant barriers to dietary adherence, with participants citing limited access to healthy food options. Cultural factors, such as traditional ingredients and norms, also influence food choices. Healthcare providers should adopt a holistic approach to support dietary adherence and improve health outcomes for individuals with type 2 diabetes in Ghana. Dieticians can teach clients about local food nutritional content, healthy cooking methods, and food storage techniques, especially in resource-constrained settings.

The use of complementary herbal products was also linked to non-adherence. Herbal products, such as cinnamon and berberine, can improve dietary management for individuals with T2D by reducing blood glucose levels [31], potentially improving glycemic control through anti-inflammatory and antioxidant effects [32]. However, their reliance may lead to decreased adherence to prescribed guidelines and medications. Poor communication between patients and healthcare providers can also hinder effective management. Misconceptions about conventional treatments [33,34] can also contribute to poor adherence. Improving communication and education is crucial for safe use.

The study highlights the need for a comprehensive approach to improve dietary adherence among individuals with T2DM in Ghana. It emphasizes the importance of targeted interventions, culturally tailored materials, and cooking demonstrations that incorporate traditional ingredients and local food preferences. It also suggests that educational interventions should be tailored to meet the needs of patients with lower education levels.

The relationship between T2D and dietary adherence is complex, requiring education and healthcare. Obese individuals have lower adherence rates, emphasizing obesity-related factors. Healthcare providers should consider BMI, comorbidities, and food availability. Collaboration between governments and healthcare systems is crucial for promoting healthy food options. A multifaceted approach, including educational interventions, policy changes, and healthcare provider support, can improve health outcomes. The study on dietary adherence among individuals with T2D in rural Ghana has both limitations and strengths. The cross-sectional design, reliance on self-reported data, and focus on a specific rural district may introduce bias and limit generalizability. However, the study contributes to the literature on T2DM management in rural Ghana, using a validated tool to assess dietary adherence and exploring factors influencing it. The findings have implications for healthcare providers, policymakers, and researchers in developing targeted interventions to improve health outcomes.

## Conclusion

Key predictors of non-adherence were gender, educational level, BMI, knowledge of T2DM, duration of T2DM, and complementary herbal use. The findings underscore the significance of environmental and socioeconomic factors, particularly food availability and accessibility, in shaping dietary choices. Community-based nutrition education, support for local food initiatives, and culturally sensitive dietary advice can empower individuals to make informed choices. Peer support groups, mobile health solutions, and partnerships with local leaders can further enhance T2DM management. To improve adherence, culturally sensitive dietary advice can be adopted by incorporating traditional Ghanaian dishes with healthier modifications, respecting cultural preferences while promoting healthier eating habits. Again, mobile health (mHealth) solutions can be utilized to deliver health information, reminders, and support to patients, given the widespread mobile phone penetration in rural Ghana. Again, local community health workers can be trained to provide basic nutrition counseling and support, leveraging existing community structures.

### What is known about this topic

- This epidemiologic transition is driven by factors like the westernization of the traditional African diet;
- Westernized diets are seen more in the urban communities than in the rural ones;
- Not adhering to the recommended diet is a principal worry for health care providers and T2DM patients, which seriously affects the disease trajectory and health outcomes.

### What this study adds

- The study presents specific data on the prevalence of dietary non-adherence among T2DM patients in a rural Ghanaian community and identifies significant predictors of it;
- Dietary counseling challenges; people living with T2DM often face difficulties adhering to recommended diets, particularly in rural areas where counseled diets may be hard to access or unavailable;
- It provides insight into the specific challenges faced by people living with T2DM in rural Ghana, highlighting the unique barriers to dietary adherence in this setting.
